# The Influence of Mixing Conditions on the Macro-Scale Homogeneity of Asphalt Mixtures Blended with Reclaimed Asphalt Pavement (RAP)

**DOI:** 10.3390/ma14154137

**Published:** 2021-07-25

**Authors:** Quan Liu, Markus Oeser

**Affiliations:** Institute of Highway Engineering, RWTH Aachen University, 52074 Aachen, Germany; oeser@isac.rwth-aachen.de

**Keywords:** reclaimed asphalt pavement (RAP), mixing conditions, macro-scale homogeneity, digital image processing

## Abstract

The homogeneity of asphalt mixtures blended with reclaimed asphalt pavement (RAP) is affected by many factors. Due to the complicated compositions of recycled asphalt mixtures, the inhomogeneity issue might cause insufficient mechanical properties of asphalt mixtures, even though a design method was appropriately adopted. Therefore, it is of great significance to study the influence of mixing conditions on the homogeneity of asphalt mixtures blended with RAP materials. This study focused on the macro-scale homogeneity of produced asphalt mixtures. Specifically, asphalt mixtures incorporated with 40% RAP content were produced in a laboratory using different mixing times and mixing temperatures. A multi-direction indirect tensile stiffness modulus (ITSM) test was proposed to quantify the homogeneity of produced samples. In addition, the digital image processing (DIP) method was used to identify the distribution of aggregates and RAP binder. The results indicated that the influence of mixing time on the macro-homogeneity of asphalt mixtures indicated that a longer mixing time was favorable for the material dispersion. The influence of mixing temperature mainly rested on two perspectives. One was that the temperature variation induced the change of binder viscosity. The other was that the temperature influences the diffusion process between RAP binder and new bitumen, which further affected the mechanical performance of produced asphalt mixtures.

## 1. Introduction

Asphalt mixtures have been extensively used in the construction of pavements. However, when pavement suffers severe distress and, as a result, no longer functions with satisfactory quality, pavement has to be reconstructed [1]. On the other hand, to address increased environmental concerns, an economic solution to deal with reclaimed asphalt pavement (RAP) is to reuse recycled materials in construction [2]. Specifically, a certain amount of RAP can be incorporated into the production of new asphalt mixtures, aiming to reduce resource consumption. Additionally, the reuse of RAP materials also has environmental benefits, as landfill usage for recycled materials can be reduced remarkably [3,4].

In the conventional production of asphalt mixtures, bitumen is evenly coated on the aggregate surface, and the aggregates are uniformly distributed, forming a relatively homogenous structure. A previous study demonstrated that the homogeneity issue played a critical role in the mechanical properties of produced asphalt mixtures [5]. Commonly, the homogeneity of asphalt mixtures is estimated by quantifying the segregation of air voids and aggregates [6,7,8]. Therefore, studies addressing the homogeneity of asphalt mixtures can be simplified as the distribution of aggregates and air voids. To date, many technologies have been developed to characterize the distribution of aggregates and air voids. Among these, digital image processing (DIP) technology is the most widespread method used to quantify the microstructure of asphalt concrete [9,10,11]. Yue et al. applied DIP to calculate the distribution, orientation, and shape of coarse aggregates in mixtures [12]. Their results indicated that major cross-sections of coarse particles preferred to lie horizontally in mixtures. Masad et al. [13] adopted X-ray computed tomography (CT) to measure air void distributions. It was reported that the air void distributions conformed to a bathtub shape, whereby larger voids existed at the top or bottom parts of a specimen. Liu et al. [14] analyzed CT images based on fractal dimension methods. Accordingly, a segregation indicator based on a fractal dimension method was proposed, which was feasible for defining the segregation of pavement. Peng et al. [7,8,9,15] conducted a comprehensive investigation of the homogeneity of asphalt mixtures. The effect of vertical aggregate homogeneity on penetration strength was studied, based on the discrete element method (DEM) method. In addition, an aggregate homogeneity index was introduced and it verified a good correlation with penetration strength. 

Due to clusters in RAP and the property variation between RAP materials and new mixtures, the segregation issue in production could possibly be more significant. Li et al. [3] adopted DIP technology to study the homogeneity of in-place recycling of asphalt mixtures. It was reported that, using DIP technology, virgin and RAP aggregate could be identified, respectively. An increase in RAP temperature and mixing time was able to improve the homogeneity of asphalt mixtures. They also concluded that the quantitative homogeneity of aggregates could control the quality of recycled asphalt mixtures. The variation in RAP materials also affected the homogeneity of asphalt mixtures produced with RAP. Vislavičius and Sivilevičius [16] studied the effect of reclaimed asphalt pavement gradation variation on the homogeneity of recycled asphalt mixtures. In that study, a stochastic simulation technology was carried out, and the simulation results claimed that the formal standard requirements were not sufficient to guarantee the homogeneity of the produced batch. Apart from the variation in RAP materials and the mixing procedure, the homogeneity issue was no longer limited to aggregates and air-void segregation; it also including binder homogeneity. Mohammadafzali et al. [17] discussed the effect of rejuvenation and aging on the binder homogeneity of recycled asphalt mixtures. It was found that a rejuvenator could be absorbed by the outer binder layers and continued to diffuse as time elapsed. 

According to the studies introduced above, it can be concluded that many factors influence the homogeneity of asphalt mixtures incorporated with RAP materials. Comparing with conventional asphalt mixtures, the homogeneity issue concerning the recycled asphalt mixtures should be extended to a multi-scale solution. On the other hand, it is known that the production of recycled asphalt mixtures continue to use conventional mixing devices, which could be unfavorable for the homogeneity of the produced asphalt mixtures. Therefore, it is necessary to study the effect of mixing, including mixing time, mixing temperature, and mixing procedure, on the homogeneity of recycled asphalt mixtures. Additionally, the homogeneity of the mix from a multi-scale perspective should be considered in the investigations. 

This study aims to investigate the macro-homogeneity of asphalt mixtures incorporated with reclaimed asphalt pavement, considering different mixing conditions. Specifically, the influence of mixing temperature and mixing time on the mechanical performance of produced asphalt mixtures will be discussed. To this end, asphalt mixtures incorporated with 40% RAP content were produced in a laboratory at different mixing times and mixing temperatures. The thermal equilibrium state was intended to eliminate the influence of thermal conductivity on the final performance of asphalt mixtures, although it deviated from actual mixing condition. In this case, the variation of macro-scale homogeneity caused by mixing could be qualified. RAP production was completed in a laboratory to avoid material variation. After producing asphalt mixtures, a multi-direction indirect tensile stiffness modulus (ITSM) test was carried out, followed by variation analyses of the obtained data. In addition, a morphological analysis of sliced asphalt mixtures was conducted, aiming to present an intuitive visualization of the morphological structures of the produced asphalt mixtures.

## 2. Materials and Experimental Methods

### 2.1. Materials

#### 2.1.1. Bitumen

**Figure 1:**
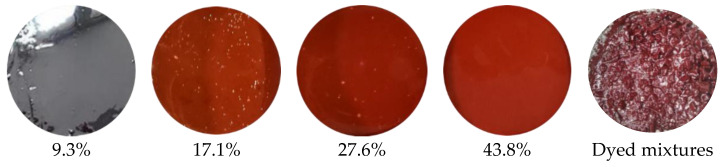
Visualization of dyed bitumen with different contents of iron oxide.

PG 70-22 bitumen was used to produce the RAP materials, and new bitumen was added to the mixing. In order to distinguish between the new bitumen and the aged bitumen in RAP materials, dyeing technology was applied for the bitumen in RAP. The dyeing technology was invented to change the color of bitumen by adding chemical additives. The use of iron oxide powder was introduced in the literature [18]. Specifically, iron oxide powder was added to the bitumen before RAP production. According to the preliminary trial, the optimal iron oxide powder was determined to be 30% by mass of bitumen, as shown in Figure 1. It could be found that when the amount of bitumen reached 30%, the bitumen could be well dyed.

**Figure 2:**
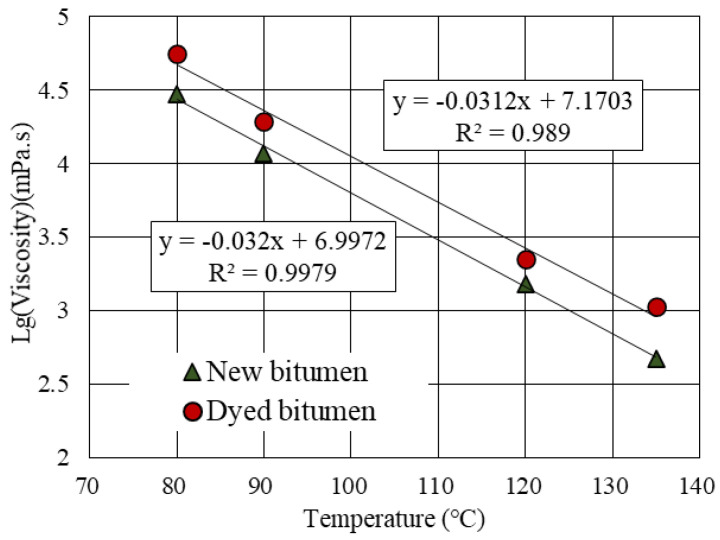
Viscosity-temperature curves for new bitumen and dyed bitumen.

Table 1 presents a conventional indicator for the new bitumen and the dyed bitumen. After dyeing, the penetration decreased and the ductility was reduced, indicating an increase in stiffness. In addition, the viscosity–temperature curves (Figure 2) [19] indicated an increase in viscosity after dyeing, while the temperature sensitivity did not change significantly.

Although the variation between the new bitumen and the dyed bitumen could alter the flowability of binders, which further affected the mixing, this study used significantly different mixing temperatures to alter the influence of mixing temperature on the macro-homogeneity of the produced asphalt mixtures. It is worth noting that the incorporation of iron oxide increased the stiffness of the produced mixtures. Therefore, the incorporated amount of iron oxide was fixed for different mixing conditions. In this case, the difference in the measured modulus was mainly ascribed to the variation of the mixing conditions. For the evaluation of the homogeneity of asphalt mixtures based on the measured modulus, the coefficient of variation (CoV) was accordingly calculated for the parallel samples. 

#### 2.1.2. Mineral Aggregate and Filler

**Figure 3:**
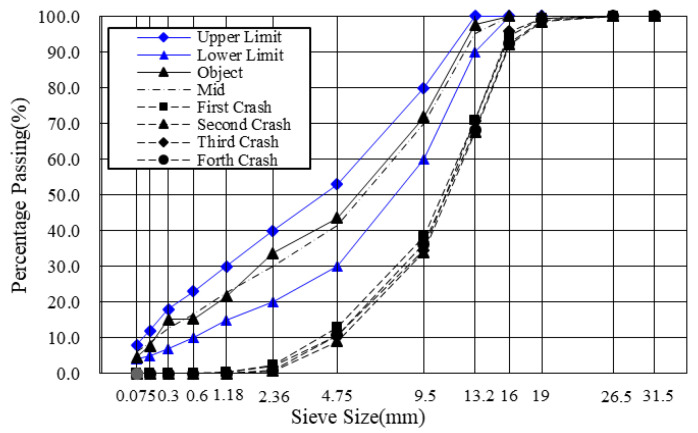
Grading curves for aggregates and crushed RAP.

This study used basalt as the aggregate and limestone as the filler. The objective gradation curve shown in Figure 3 is in line with the gradation of AC-13 from the specifications [23]. The binder was fixed at 4% for the production of mixtures, and the filler–binder ratio was 1.0 in this study.

#### 2.1.3. RAP Preparation

**Figure 4:**
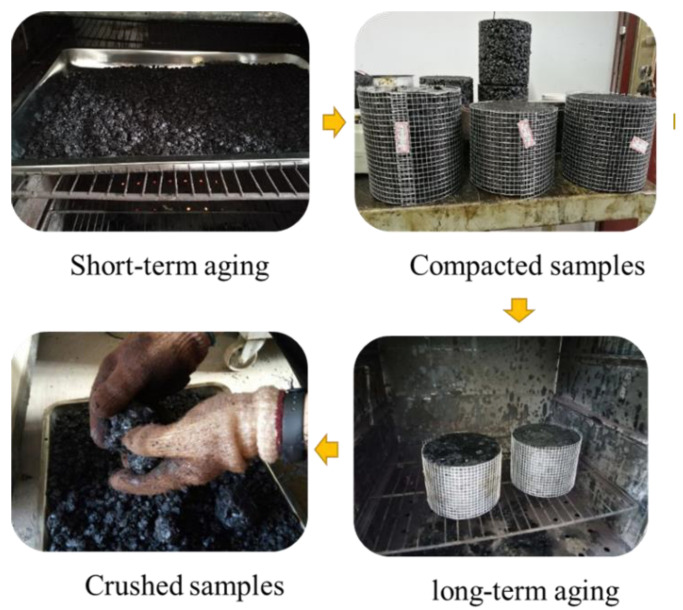
The process illustrating RAP production.

Following the gradation used in this study, sufficient asphalt mixtures were mixed with 4% binder content. In what follows, part of the asphalt mixtures was subjected to SHRP short-term aging (at 135 °C for four hours) in a loose state [1]. After that, the short-term aged loose asphalt mixtures were compacted into cylindrical specimens (150 mm diameter, 115 ± 5 mm height, and 4.5% target air void) using a Superpave Gyratory Compactor (SGC) [24]. The compaction parameters were set to 1.25° (angle of gyration), 0.6 MPa (vertical pressure stress), and 30 rpm (gyration speed). The RAP materials were then produced after applying SHRP long-term aging (at 85 °C for 5 days) on the compacted asphalt mixtures with at least 1 day at ambient temperature [1]. The variation of gradations for the RAP materials was examined as the indicator to control the material homogeneity. Specifically, four samples were heated to 135 °C for four hours and crushed into pieces. Four samples were randomly taken from the crushed RAP and subjected to gradation tests, the results are shown in Figure 3. It can be seen that the grading results are very close to each other. The whole process is illustrated in Figure 4.

### 2.2. Experimental Program

#### 2.2.1. Fabrication of Specimens Containing RAP

The new aggregate was heated to 150 °C for eight hours to eliminate moisture to avoid thermal conductivity. The virgin bitumen was firstly heated to 135 °C to obtain sufficient flowability. The crushed RAP materials and new aggregates were preheated to targeted temperatures (90, 110, and 130 °C) for two hours. In order to produce the asphalt mixtures containing the RAP, the crushed RAP materials and new aggregate were firstly blended using a mechanical mixer. After that, flowable bitumen was added to the blend for a specific amount of time (30, 60, 120, and 240 s). The proportion of RAP was maintained at 40%. In this case, 12 kinds of mixing conditions were considered in the production of recycled asphalt mixtures. Four cylindrical specimens (with 150 mm diameter and 115 ± 5 mm height) were compacted using a gyratory compactor for each case. The specimens were conditioned at room temperature for one day and then cored into cylindrical specimens with a diameter of 100 mm. After that, two specimens were cut into two cylindrical specimens with a height of 40 mm, and another two specimens were sliced into 10-disc specimens with a thickness of 20 mm.

#### 2.2.2. ITSM Tests

**Figure 5:**
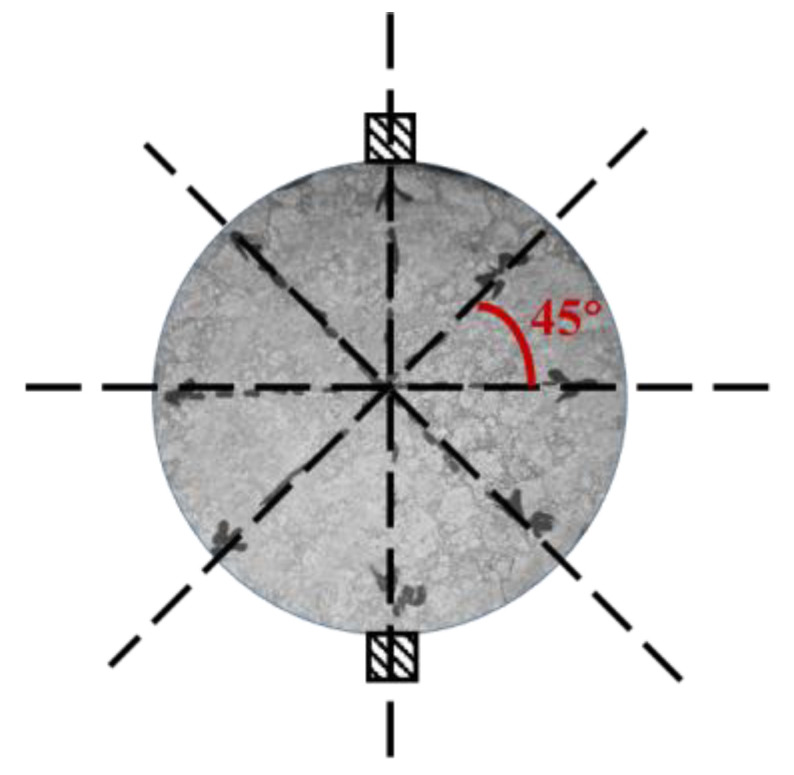
ITSM test from four directions.

As shown in Figure 5, the ITSM test was applied to two specimens from four different directions at 20 °C using UTM-25. The test was carried out following the standard for indirect tensile tests for the resilient modulus of bituminous mixtures [25]. In this case, eight ITSM values for one mixing condition could be obtained. Based on the variation analysis on the eight ITSM values, the macro-homogeneity of the asphalt mixtures in terms of mechanical performance could be quantitatively estimated. Herein, this study defined macro-homogeneity as the overall performance of the asphalt mixtures. Corresponding micro-scale homogeneity was defined by the migration and diffusion behavior of binders in the asphalt mixtures.

#### 2.2.3. Color Image Analysis

High-quality pictures were taken for both sides of each disc specimen using a digital camera with carefully controlled lighting and exposure conditions. It was found that the binder was easier to identify when the specimen was wetted. Therefore, water was sprayed before pictures were taken. In total, 240 pictures of the specimens were collected.

The obtained 240 images were subjected to the image analysis using MATLAB, which conformed to the following steps.

**Step 1**: Preliminarily enhance the image quality: Image contrast is enhanced using the histogram equalization function. The histogram equalization method processes the image to adjust the contrast of the image by modifying the intensity distribution histogram. The purpose of this technique is to give an image of the cumulative probability function related to a linear trend.

**Step 2**: Calculate sample colors in L*a*b color space for each region: for the image segmentation, the RGB image was firstly convert to the CIE L*ab space. CIE L*ab was defined by the lightness and the color-opponent dimensions a and b. The CIE L*ab space of an image is usually calculated for specific purpose, such as the image segmentation. 

**Step 3**: Classify each pixel using the nearest neighbor classification: By calculating the Euclidean distance between that pixel and each color marker. The smallest distance could determine that the pixel most closely matched that color marker.

**Step 4**: Display results of nearest neighbor classification: The label matrix contains a color label for each pixel in the image. Based on this, the label matrix was calculated to separate objects in the original image by color.

**Step 5**: Convert each classification region into binary images.

**Figure 6:**
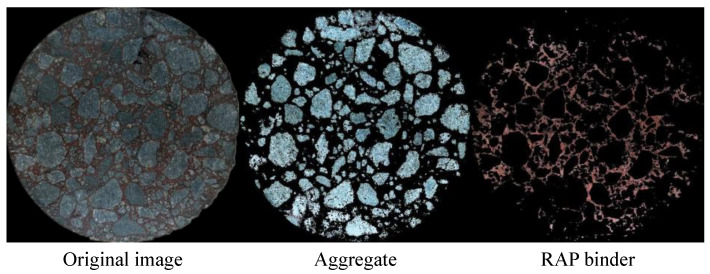
Montage of original image, aggregate, and RAP binder.

Following the above steps, the aggregate component and RAP binder component can be successfully identified and segmented, as shown in Figure 6.

## 3. Results and Discussion

### 3.1. Macro-Scale Homogeneity Based on ITSMs

**Figure 7:**
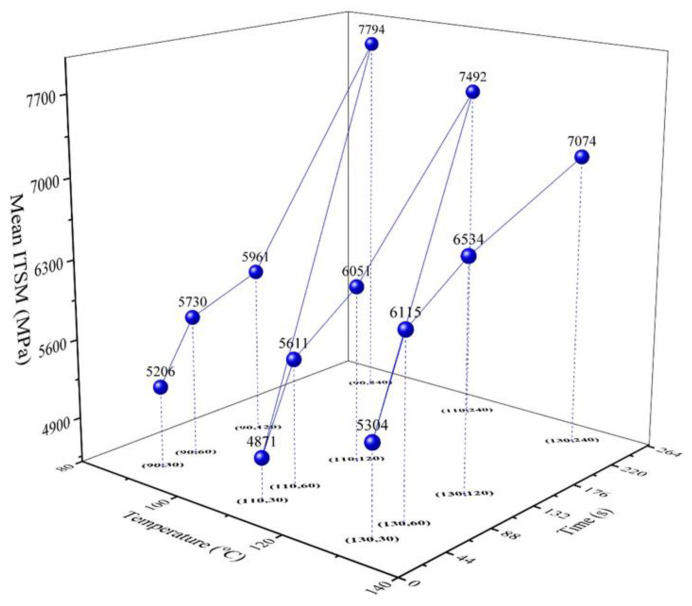
The average ITSM versus mixing temperature and time.

According to ITSM measurements, the mean value, standard deviation (SD), and coefficient of variation (CoV) of ITSMs were calculated for each mixing condition. Figure 7 shows the mean values of the ITSM for all cases. It could be found that the mixing conditions significantly influenced the stiffness of the asphalt mixtures. Therefore, it is essential to use appropriate temperature and time parameters, regardless of the recycling types. It is worth noting that the maximum mean value of ITSM (7794 MPa) was observed when the mixing temperature and time were 90 °C and 240 s, respectively. In previous studies, the increase of RAP content increased the stiffness of asphalt mixtures due to aged bitumen in the RAP materials.

Similarly, the high stiffness obtained at a low temperature might be attributed to the low blending degree between the aged bitumen and the new bitumen. In contrast, mixing the asphalt mixture at high temperatures accelerated the diffusion between the aged bitumen and the new bitumen. Hence, a high stiffness modulus was observed at low temperatures. 

The minimum mean value of ITSM occurred at a temperature of 110 °C and a mixing time of 30 s. This observation indicated that the influence of temperature on the stiffness modulus of the asphalt mixtures was not monotonic. Therefore, a critical temperature was most unfavorable for forming asphalt mixture stiffness, for instance, 110 °C in this study. The mixing time benefited the formation of asphalt stiffness. There are two plausible reasons for this. With the increase in mixing temperature, the bitumen binders suffered secondary aging. Additionally, a longer mixing time made it easier to achieve a better morphological distribution, although morphological distribution on the stiffness formation requires further investigation.

#### Effect of Mixing Time on Stiffness

**Figure 8:**
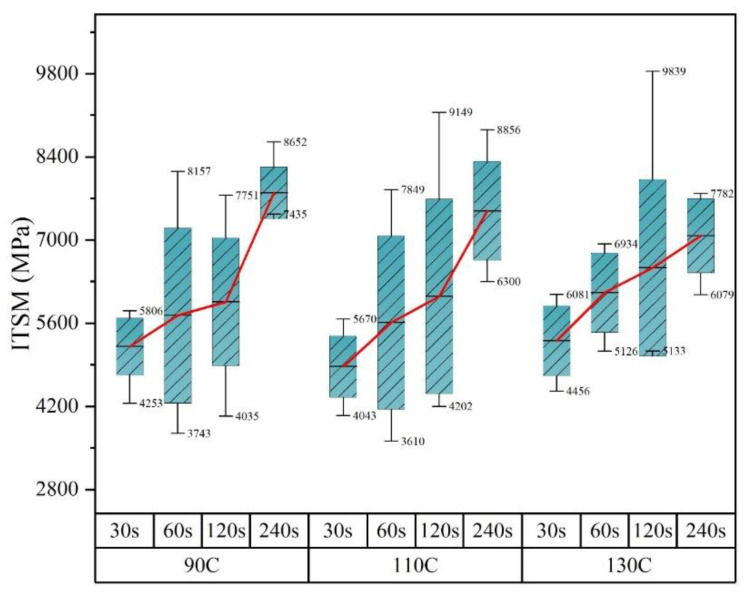
The stiffness values versus mixing time at different mixing temperatures.

**Figure 9:**
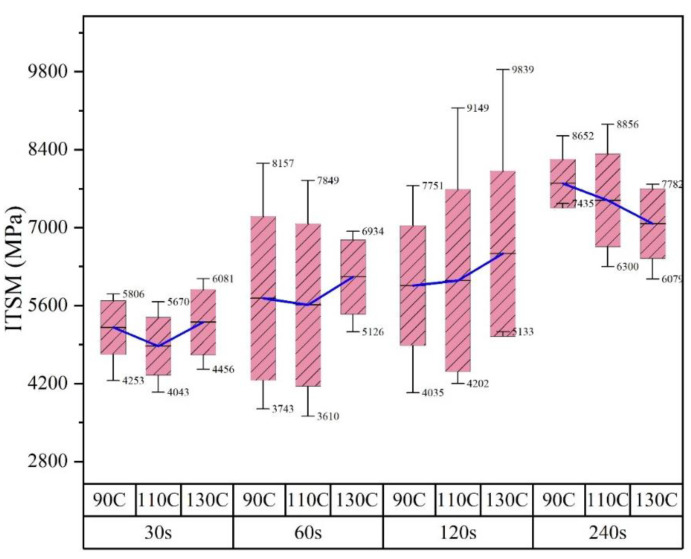
The stiffness values versus mixing temperature at different mixing times.

This section discusses the influence of mixing time on the stiffness of asphalt mixtures. Figure 8 and Figure 9 interpret the same results in two different presentations for the benefit of analysis. Figure 8 shows the trend of stiffness modulus as the mixing time increased. It can be seen that, at all temperatures in this study, the average stiffness modulus increased progressively when extending the mixing time from 20 s to 240 s, which indicated that the extension of mixing time positively influenced the stiffness formation of asphalt mixtures containing RAP. In the mixing process, particles included in both new aggregates and RAP experienced complicated movements, during which time, the RAP clusters broke and simultaneously mixed with new aggregates. Therefore, the extension of mixing time was favorable for achieving a homogenous aggregate skeleton of asphalt mixtures. The increase in the modulus was also ascribed to the secondary aging of binders stated above.

Apart from the average ITSMs, the error bar and value boundary of ITSMs measured at specific mixing conditions are shown in Figure 9 to estimate the data variation. At 90 and 110 °C, the maximum standard error and data difference were observed when the mixing time was 60 s, indicating the worst homogeneity of the specimens. While at 130 °C, the worst homogeneity of the asphalt mixtures happened at 120 s. In conclusion, the homogeneity of the asphalt mixtures first increased then decreased with mixing time. In addition, the increase in temperature prolonged the mixing time when the worst state took place. In the beginning, the clusters could not be crushed in time and, therefore, acted as large particles existing in the asphalt mixtures after compaction. The asphalt mixtures produced with a short mixing times were deemed to be in the momentary homogeneity state. When the mixing continued, the clusters would be crushed under external mechanical forces and progressively blended with newly added mixtures. Immediately after the breaking of clusters, the momentary homogenous state disappeared until a further mixing effort was applied. This assumption perfectly explained the homogeneity evolution of the asphalt mixture with mixing time. As for the influence of temperature, the termination of the momentary state prolonged as the cluster breaking could significantly interfere with binders’ viscosity variations at different temperatures.

(1) ***Momentary homogeneity with insufficient temperature***

As stated above, when the mixing time was very short, the produced asphalt mixture might achieve a momentary homogeneous state due to the clusters in RAP not being wholly crushed under external forces. In such a state, the aged bitumen in RAP and new bitumen were separated with limited interactions. Therefore, the diffusion process between them was limited. In this case, the temperature dominantly contributed to the flowability of aggregates both in RAP and in the newly added ones. Within an insufficient temperature range, the increase in temperature increased the flowability of particles. However, this improvement negatively influenced the breaking of clusters in RAP. As a result, the stiffness decreased, although the temperature increased.

(2) ***Momentary homogeneity with sufficient temperature***

If the temperature continued to increase to a sufficient range, the clusters would be softened and would lose the adhesive force inside. In this case, the temperature played a positive role in the breaking of clusters in RAP. The momentary homogeneous state would shift into an unbalanced state when the blending degree of RAP and new aggregates started to grow. Therefore, the stiffness modulus increased when the temperature was at 130 °C, as seen in Figure 9.

(3) ***Near-real homogeneity with insufficient temperature***

Extending the mixing time is helpful for the formation of real homogeneity in asphalt mixtures. The mixing time and temperature mutually determined the homogeneity state of asphalt mixtures. As seen in Figure 9, when the mixing time was 120 s, the asphalt mixture experienced a change from a momentary homogeneous state to a real homogeneous state called a near-real homogeneous state in this study. In such a state, the temperature played two roles. One rested on the speed of the state shift. The other one contributed to the diffusion between new and aged bitumen. The role of temperature concerning the state shift was more explicit than the diffusion.

(4) ***Real homogeneity with sufficient temperature***

When the mixing time was 240 s, the asphalt mixtures almost achieved real homogeneity since the error bars were small. In this condition, the temperature mainly contributed to the diffusion between aged bitumen and new bitumen. With the increase in temperature, the diffusion rate would be accelerated, during which the aged bitumen would be softened, inducing a decrease in the stiffness modulus.

**Figure 10:**
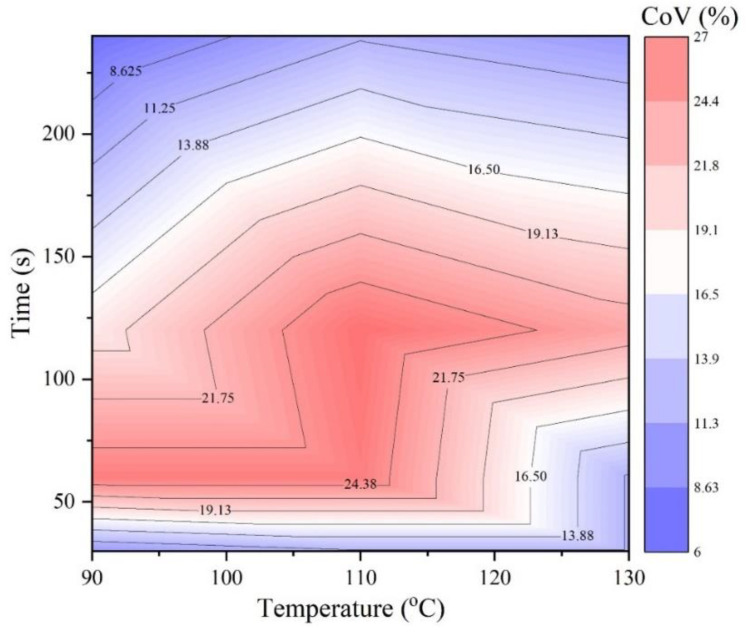
The stiffness variation caused by mixing time and mixing temperature.

Figure 10 presents the calculated coefficient of variation (CoV) for different mixing conditions. The CoV was used herein to evaluate the homogeneity of the asphalt mixtures. As seen in Figure 10, the red areas indicated the inhomogeneous state, while the blue ones referred to the homogeneous asphalt mixtures. It should be noticed that Figure 10 cannot tell the difference between momentary homogeneity and real homogeneity. When the mixing time was from 0 s to 30 s, momentary homogeneity was observed. Furthermore, when the temperature increased to 125 °C, the momentary homogeneity state would last a long time, extending to around 75 s. The real homogeneous state took place when the mixing time was beyond 150 s for a low temperature (90 °C) and 175 s for a high temperature (135 °C). 

The inhomogeneous state occurred in the rest area. It can be seen that the combination of temperature (110 °C) and mixing time (120 s) was the most unfavorable mixing condition in terms of the homogeneity. Additionally, the inhomogeneous state area was shown in a ‘Z’ shape. The most inhomogeneous state can be achieved quickly at a low temperature or a longer time at a high temperature. 

It is believed that Figure 10 can be improved further if more and more mixing conditions could be considered in one research study. This construction of a homogeneity pattern as a mixing time and temperature function can efficiently guide the mixing procedure in practice.

### 3.2. Visualization of the Homogeneity of Asphalt Mixtures

**Figure 11:**
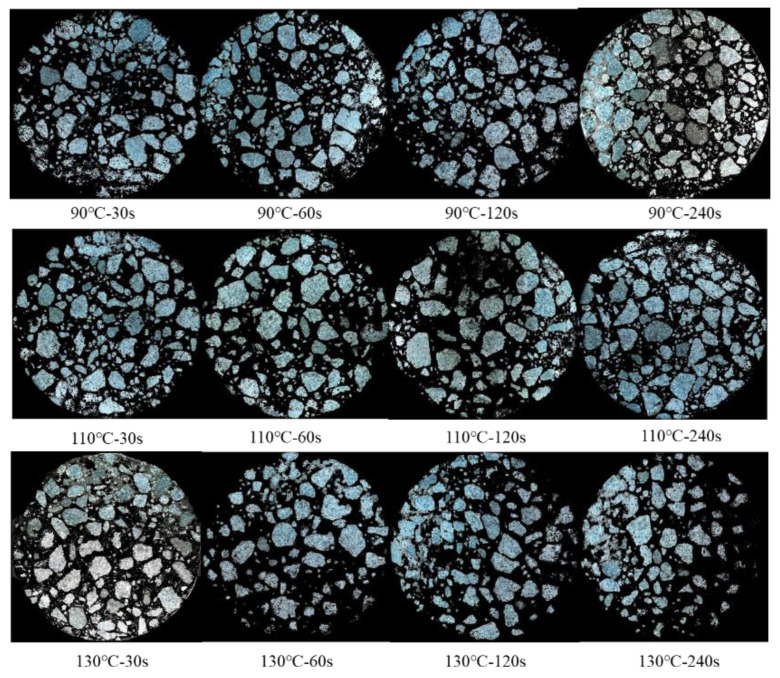
Visualization of aggregate distribution at different mixing conditions.

In total, 240 images were subjected to image processing to identify aggregates and RAP binder morphologies, respectively. Figure 11 presents one image for each mixing condition. Significant variation among the 12 images was not observed. This indicated that, for a conventional asphalt mixture, 30 s was sufficient to disperse the aggregate, forming a typical asphalt mixture structure. This complied with actual situations since mixing, in practice, was usually controlled within 1 min. 

It can be seen that it is not possible to tell the difference caused by the mixing condition. The primary reason for this was that the aggregates in RAP or newly added aggregates could not be further distinguished. From another perspective, aggregates in RAP had identical properties to the newly added ones. Given that the binders were well coated on the aggregate surface, the mechanical performance of asphalt mixtures was mainly determined by the skeleton structure of aggregates, which was not a critical problem with a well-designed mixing procedure. 

Another limitation of examining the aggregate distribution shown in this study was that the dimension reduction from 3D to 2D might lose considerable structural information. Therefore, it is not recommended to evaluate the homogeneity of asphalt mixture by visualizing aggregate morphology. 

**Figure 12:**
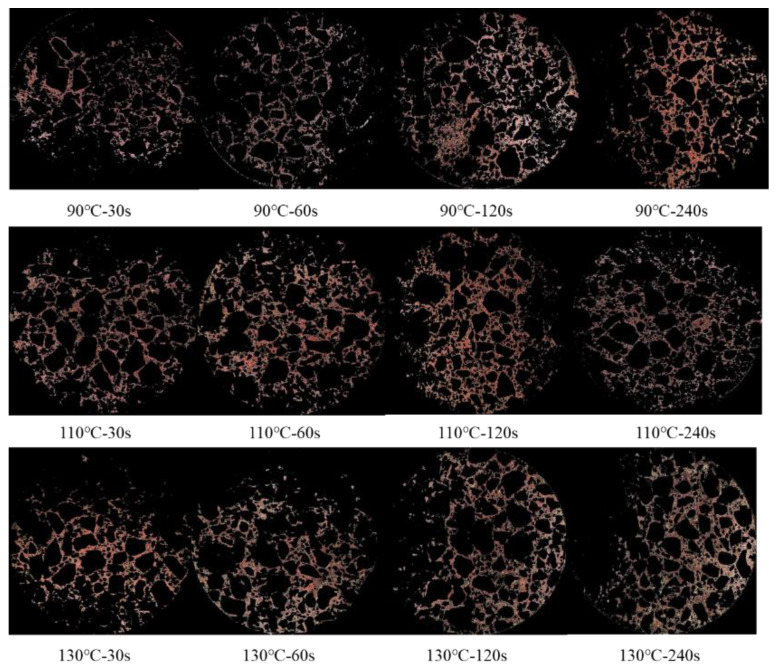
Visualization of RAP binder distribution at different mixing conditions.

Figure 12 shows a visualization of RAP binder morphology. The red area indicated the place where the RAP binder was located. It can be seen that the RAP binder, even with the mixing time of 240 s, can be dispersed uniformly in the asphalt mixtures. Based on the visualization of the RAP binder, it is not easy to achieve a complete mixing even the mixing time was 240 s and the temperature was 130 °C. Additionally, it can be observed that there is no significant rule for the RAP binder pattern when the mixing conditions varied. However, it is clear that when the mixing time is short (30 s or 60 s), the phenomena “partial missing of RAP binder” quickly occurred, which demonstrated the presence of RAP clusters. 

When the temperature was 90 °C, a relatively homogenous distribution of RAP binder was observed at 120 s. According to the last section’s analysis, the RAP binder’s viscosity was high, and the migration of RAP binders was mainly attributed to external force. Thus, extending the mixing time was beneficial to the migration of RAP binders. It is worth noting that the RAP binder was not detected in some parts of the image at 240 s, indicating that the cluster of new aggregate and new bitumen also occurred in the mixing process. Similar phenomena were observed at other mixing temperatures.

## 4. Conclusions

Based on the results and discussion in this study, the following conclusions can be drawn:

(1) The macro-homogeneity of asphalt mixtures is associated with mixing temperature and mixing time. The asphalt mixtures produced with different mixing conditions show distinct mechanical performance. 

(2) A long mixing time benefited the distribution of aggregates in both RAP materials and new mixtures. The increase of mixing temperature induced binder flowability, which improved the migration and diffusion of binders. According to the results, the influence of mixing time and temperature should not be determined separately. An optimal combination of mixing time and temperature exists to achieve a satisfying mechanical performance of asphalt mixtures containing RAP materials.

(3) Although many efforts have been made to repeat the production of asphalt mixtures with RAP materials in the laboratory. The addition of iron oxide into bitumen significantly changed the binder properties. Therefore, it should be promising to use other technologies to identify the difference between RAP materials and newly added mixtures. The laboratory investigations provided a basic understanding of the macro-homogeneity of asphalt mixtures with different mixing temperatures and mixing times. However, in situ mixing procedures are different from laboratory ones. For a deep understanding of the homogeneity of asphalt mixtures containing RAP materials, carrying out a full-scale investigation in the field is recommended.

## Figures and Tables

**Table 1:** Empirical parameters of PG70-22 bitumen and the dyed binder.

	Penetration @25oC (dmm) [20]	Softening Point (°C) [21]	Ductility @15 °C (cm) [22]
New bitumen	68	49.2	158.4
Dyed bitumen	52	49.7	26.4

## Data Availability

All data used during the study appear in the submitted article.
